# Trends and prospects of geothermal energy as an alternative source of power: A comprehensive review

**DOI:** 10.1016/j.heliyon.2022.e11836

**Published:** 2022-11-25

**Authors:** Mohammad Towhidul Islam, M.N. Nabi, M.A. Arefin, K. Mostakim, Fazlur Rashid, N.M.S. Hassan, S.M.A. Rahman, S. McIntosh, B.J. Mullins, S.M. Muyeen

**Affiliations:** a Georgia Southern University, Statesboro, GA 30458, USA; b Central Queensland University, WA 6000, Australia; c Safe and Reliable Nuclear Applications-Nuclear Reactor Operation and Safety, LUT University, 53850 Lappeenranta, Finland; d Rajshahi University of Engineering and Technology, Bangladesh; e Missouri University of Science and Technology, Rolla, USA; f Central Queensland University, Cairns, QLD 4870, Australia; g BERF, Queensland University of Technology, Brisbane, Australia; h School of Environment Science and Engineering, Southern Cross University, Lismore, NSW, Australia; i Curtin University, WA, Australia; j Qatar University, Qatar

**Keywords:** Geothermal potential, Direct and indirect use, Power generation, Techno-economic analysis, Bound of applicability

## Abstract

The world has capitalized on numerous renewable energy resources by developing its energy infrastructure mainly around solar, biomass, and hydro energy. However, geothermal energy has not yet been developed at a significant scale, despite reports from 62 wells showing evidence of geothermal gradients ranging from 20.8 °C/km to 48.7 °C/km in various areas of the world. Recent studies suggest that Bangladesh also has a huge potential for geothermal energy. This review extensively reports on exploiting the range of geothermal temperature in various direct and indirect energy application sectors including but not limited to the agriculture and industrial sector of Bangladesh. Additionally, the authors have analyzed and proposed adaptable measures to harness the abundance of geothermal energy. Furthermore, a comparative and possible solution has been discussed extensively for implementing a geothermal powerplant by analyzing techno-economic costs, policies, and systems of other countries in the world. Further, this review also shows the prospect of geothermal energy for Bangladesh as a case study.

## 1 Introduction

**Figure 1:**
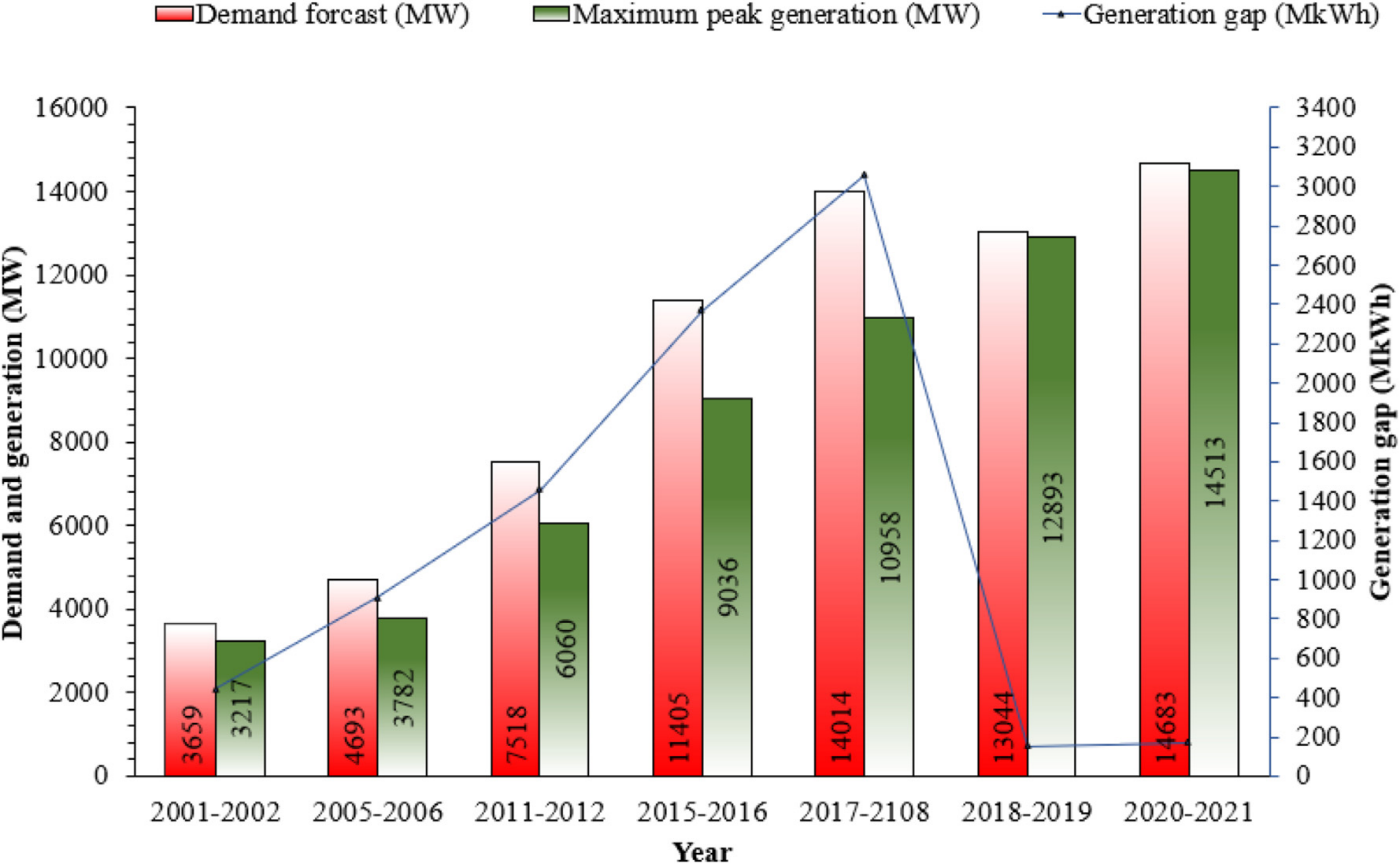
Demand and generation gap of electricity in the perspective of Bangladesh [2, 3].

Among numerous factors, the steady increase in energy production has played a prominent role in global development. Research into economics has identified a correlation that a nation's gross domestic product (GDP) is commensurate with its ability to use energy [1]. However, global energy demand solely depends on fossil fuels. Over-reliance on fossil resources increases economic, societal, and environmental risks throughout the world. Besides, the present scenario of electricity production is unable to meet increasing demand. Hence there persisted in a generation shortfall as seen in Figure 1 (a case study in Bangladesh), which must be met either by importing or utilizing the available resources more wisely [2]. It is seen from Figure 1 that the demand forecast is higher than the maximum peak generation though both the two are increasing with time. However, according to the statistics of previous years, the extrapolated data in the year 2020–2021 shows that there will remain a generation gap in the upcoming years. Even the gap may increase due to the rapid growth of industrialization and populations.

**Figure 2:**
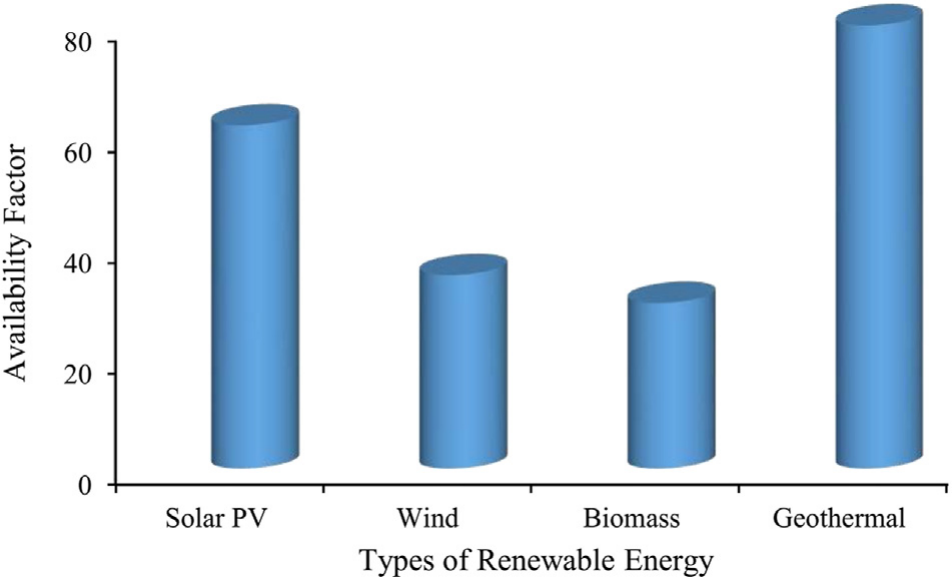
Reliability of renewable energy sources [4, 5].

In this circumstance, the effective utilization of renewable energy can create a sustainable energy future globally. Figure 1 shows that the demand forecast (14683 MW) and maximum peak generation (14513 MW) in the year 2021–21 are close to each other. Therefore, if renewable sources like geothermal energy can be added as a potential source of energy then the generation gap of around 500 MW can be minimized. However, establishing power production around solar, wind, hydro, and biomass is weather-dependent and so it is inefficient. On the other hand, geothermal energy is not completely weather dependent, while its generation capacity is very high. Hence, the addition of geothermal energy with the conventional grid system can reduce purchasing price of energy per unit area which eventually improves the GDP of a country [1]. Conversely, a country's development lies in its uninterrupted energy generation with high efficiency. Therefore, to aggregate continuous power with national energy generation using renewable resources, a promising solution is required. In this concern, geothermal energy utilization may be an option as it totally weathers independent and has the highest availability factor than of other renewables (Figure 2) [4]. Since the heat inside the earth's surface will not be depleted and can ensure energy consistently at high efficiency with minimal environmental impact.

Despite the world having significant potential geothermal resources, limited effort has been expended towards development. Moreover, any significant steps have not been taken to establish a framework for exploring geothermal energy. In the case of Bangladesh, governmental organizations such as the Ministry of Water Resources and the Ministry of Environment have conducted a geological survey to identify the geothermal potential. Also, one private organization, Anglo MGH Energy, has invested in developing a geothermal power plant with a capacity of 200 MW [5]. The project will generate steam power from a hydrothermal basin near Salander in the district of Thakurgaon. However, this facility is still in the planning phase.

However, geothermal energy harvesting is still in its early stages compared to other energy sources, rendering it difficult to adopt field investigation for evaluating these resources and their suitability. Only a few studies have reported the potentiality of geothermal energy but have fallen short of detailing possible applications and processes suitable for the world [6, 7, 8]. This way it would help to fulfill the high pressure of future energy demand as the demand for global energy is increasing significantly [9, 10]. Thus, an extensive evaluation of potential geothermal resources and a pathway to efficient utilization is required.

Previous research didn't show the prospects of geothermal powerplant based on effective cost analysis, feasibility study, environmental concerns, comprehensive proposal, and so on [8]. Therefore, this review discusses and analyzes the potential of geothermal energy as an alternative energy source based on the geothermal powerplant, feasibility concerns, and environmental impacts globally. The authors have extensively reviewed the research to date on geothermal energy in the world. However, as there is limited peer-reviewed literature regarding the potential and utilization of geothermal energy, the authors have included information from media reports, reports provided by the governmental organizations and recognized magazines. Based on the data and aggregated information, this review demonstrates a comprehensive proposal for incorporating the abundance of geothermal energy globally into a future renewable energy matrix. Additionally, the review also reflects on the established methods of converting geothermal energy around the world, to recommend effective processes of utilizing the geothermal energy from exploration to economic extraction. Furthermore, based on geographical status and economic condition, a comprehensive understanding of developing the most favorable types of geothermal power plants for Bangladesh are discussed. However, in this review, geothermal energy potential is discussed in section 2, technologies to convert geothermal energy in section 3, a comprehensive proposal in section 4, management model in section 5, geothermal plants in section 6, cost analysis in section 7, and application in section 8. This review concluds a much more prominent opportunity for further implementation of geothermal-related projects. Therefore, the authors have suggested effective ways to move forward with the creation of geothermal energy infrastructure along with their bounds of applicability. Also, in the wake of fossil fuel, world will face unimaginable backlash from its people in need of energy. Renewable energy is the most sustainable and dependable way to face an impending energy crisis. Incorporating geothermal energy within the existing renewable energy sector is predicted to be greatly effective in the long run. To generate power production from renewable resources, this review will provide valuable guidelines for global policymaking.

## 2 Geothermal energy potential

Renewable energy sources including geothermal energy can be integrated into the grid to implement hybrid energy systems that will help to mitigate the high demand for energy with low cost of energy (COE) and net present cost (NPC) [11]. Eventually, it will reduce the overall COE and so even the poor people can get access to electricity. Consequently, GDP will increase for developed and developing countries like Bangladesh. Economically, if geothermal powerplant can be set up then more unemployed people can get opportunities for work. In rural areas, people will also be benefitted from this source of energy by resolving their energy demand and unemployment problems. On the other hand, environmentally, geothermal energy is viable for Bangladesh as it generates less environmental pollution, harmful wastes, radioactive materials, decay materials, and so on.

**Figure 3:**
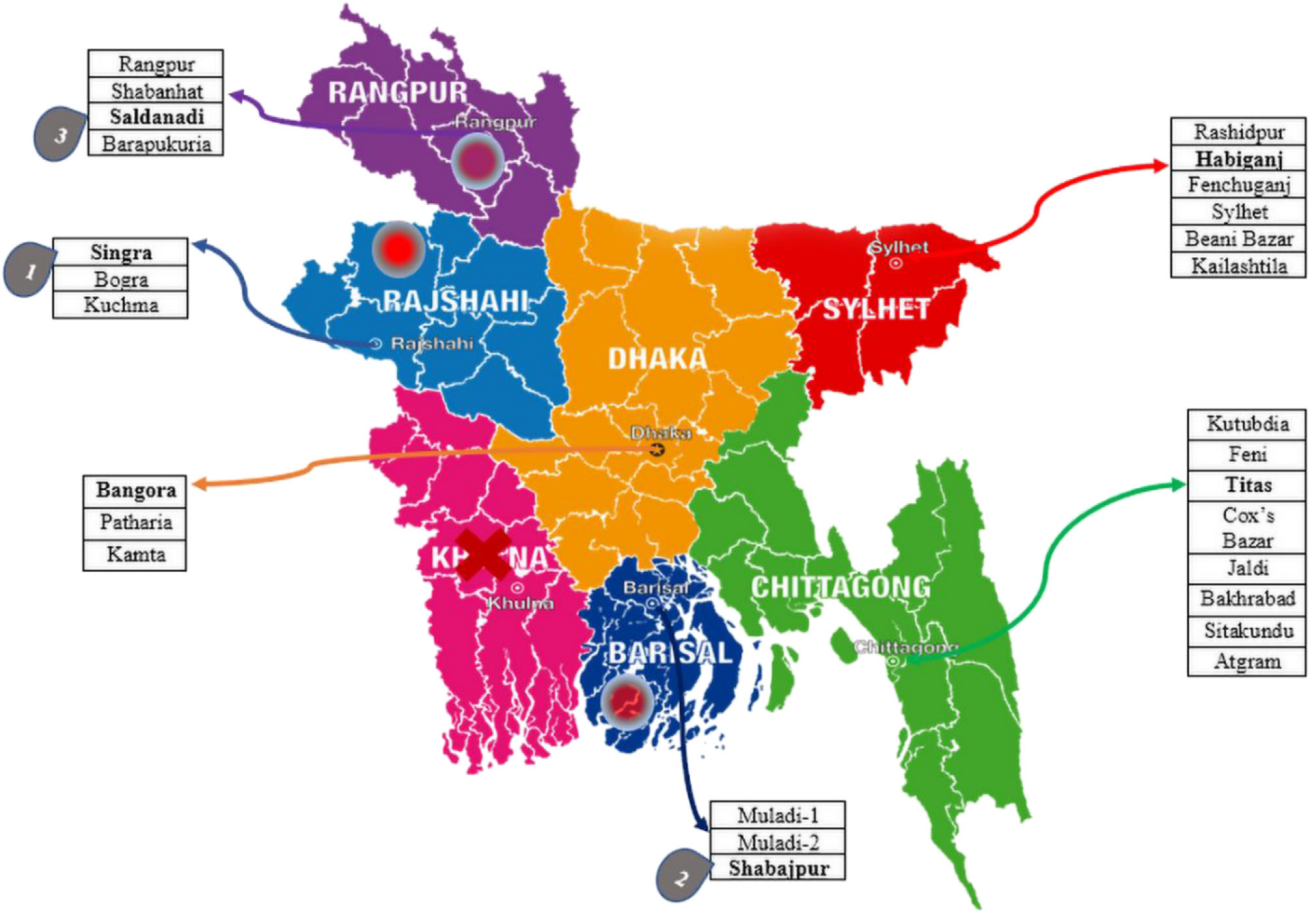
Graphical representation of potential locations in the northwest and southwest regions in Bangladesh.

However, geothermal energy is one of the most powerful, natural, and renewable sources of energy globally [12]. From the slow decay of radioactive particles in rocks, high temperatures are generated inside the earth which is a significant source of energy [13]. In the case of developing countries like Bangladesh, renewable and conventional fuels are significant sources of future energy [14]. Figure 3 illustrates the graphical representations of different potential locations for geothermal energy in northwest and southeast regions in Bangladesh. These potential locations are divided into 6 divisions, where the highlighted locations represent the highest potential of each division. However, the northwest having different geological aspects such as the hydrogeological settings, seismicity and earthquakes, cluster of basement faults and surface thermal anomalies indicate the existence of potential geothermal reservoirs at only a depth of a few kilometers below the earth's surface. The geothermal gradient in this region ranges from 20.8° to 48.7 °C/km [23]. Studies have shown the potential geothermal energy in Thakurgaon, due to the presence of some thermal manifestations and related evidence of the existence of some shallow aquifers [15]. The geothermal energy potential of these areas was also demonstrated by the work of Mizanur Rahman [15]. Moreover, in the Bogra shelf region, Singra-Kuchma-Bogra is a promising and potential area [9]. The Singra well, with over 150 °C bottom hole temperature holds the most auspicious position among the three areas [9]. Barapukuria coal basin and the Madhyapara hard rock mine area are two other potential zones for geothermal energy, among which the latter was found with a temperature variation in the range of 67°–153 °C, indicating the presence of a low-temperature geothermal reservoir in the area [16]. Moreover, it is found that the temperature at 2500 m below the earth's surface in the northwestern tip of Bangladesh, near Salbanhat-1 well, was 79 °C and at a depth of 4,000 m it increased to 110 °C [17]. Also, the GDH-65/11 well recorded 47 °C at a depth of 587m [14].

**Figure 4:**
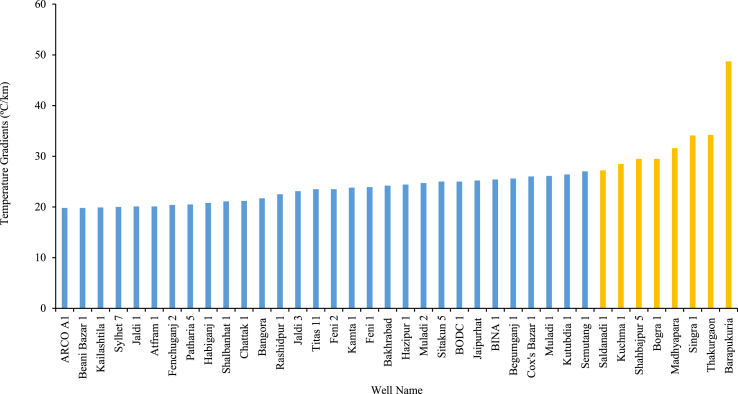
Geothermal gradients at different locations in the northwest (yellow) and southeast (blue) regions of Bangladesh [9, 18, 19].

Also, the geothermal gradients at different locations in the northwest and southeast regions are shown in Figure 4 from which a comparison can be obtained between these two regions. The southeast region has a geothermal gradient from 19.8° to 27 °C/km. Based on the presence of some thermal springs, the Sitakund hilly area has become a place of interest. From Figure 4, the highest gradient in the southeast is 27 °C/km in the Semutang-1 well located in Khagrachari. In general, the geothermal gradients in the northwest suggest more favorable conditions compared to the southeast. The wells at Thakurgaon and Barapukuria show the highest temperature gradients with values of 34.2 °C/km and 48.7 °C/km, respectively.

**Figure 5:**
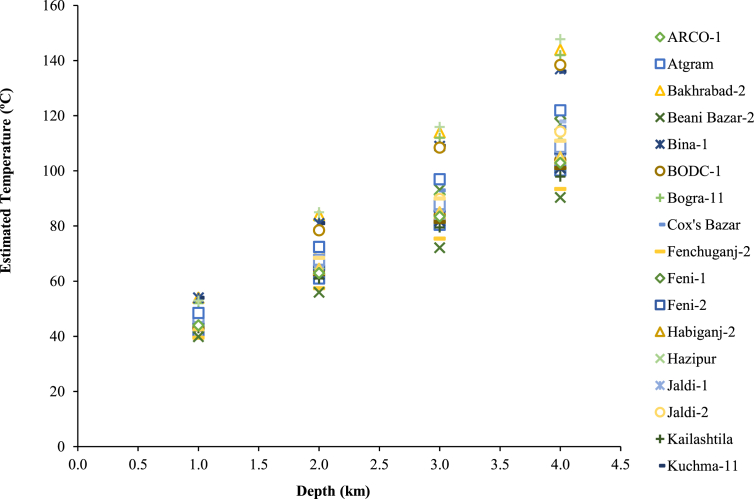
Subsurface temperature distribution in the southeastern basin of Bangladesh.

However, the subsurface temperature distribution at different depths leads to the feasibility analysis for energy extraction from geothermal resources [16]. Figure 5 illustrates the subsurface temperature distribution of different areas in southeast regions [20, 21]. The depth versus temperature is roughly linear, so the temperature according to depth can be calculated and can be used to design a suitable energy extraction process in these sites. For example, it is reported [21] that the temperature required for the dry steam and flash steam types of power plants is recommended to be >150 °C and for the binary cycle power plant the required temperature can be <100 °C which is discussed further in this study. Altogether this information supports the presence of several suitable geothermal sites across Bangladesh.

### 2.1 Favorable geothermal sites

In the case of Bangladesh, the delta system developed by two rivers, the Ganges and the Brahmaputra is considered one of the largest deltas in the world [22]. This delta supplies a large amount of sediment from the Himalayas. However, Bangladesh is situated in the northeast Indian plate closed to the edge of the Indian craton. The tectonic features of Bangladesh are dominated by a huge geosynclinal basin and a stable pre-Cambrian platform. It is further separated by a narrow northeast-southeast trending hinge which is also known as Palaeo continental slope. According to Guho et al. [7], the geological map of Bangladesh can be divided into five divisions. They are Sub-Himalayan foredeep, Bogra slope, Folded belt, Deep Sedimentary Basin, and Garo-Rajmahal Gap. These five divisions are further divided into different subdivisions which are summarized with their special features in Table 1 according to the temperature gradients and the ranges of temperature at different depths. Among the different regions, the Sub-Himalayan foredeep shows relatively low-temperature gradients which exhibits less feasibility for geothermal energy extraction. On the contrary, the Garo-Rajmahal gap and Rangpur Saddle show comparatively higher potential for energy extraction. This is because of having included the northern slope of Rangpur Saddle with Himalayan Foredeep [23]. Moreover, at a depth of 80 m in Thankurgaon region exhibits the warm water up to 36 °C, which shows the potentiality for installations of geothermal energy harnessing systems [15]. However, the high surface temperature distributions can also lead to a drilling dept at a reasonable cost, which shows the feasibility of implementing a geothermal power plant. It can be seen from the regions of Bogra slope and Folded belt basin that the subsurface temperature at a depth of 1–4 km varies within 50–150 °C which is also can be a part of geothermal resources. Though the temperature gradients in the Folded belt are relatively lower than Rangpur Saddle and Bogra slope this region is more favorable than the Deep sedimentary basin regions which include Sylhet, Mymensingh, Pabna, Barisal, Madhupur, Faridpur, and Chandpur. These regions have less feasibility for geothermal energy extraction.

**Table 1:** Location and temperatures recorded at the various geothermal site.

Division	Subdivisions	Temperature gradient	The temperature at variable depth (km) (1–4 km)	Comments	References
Sub-Himalayan foredeep	Northern slope of Rangpur saddle	21.1 °C–79 °C	2.5 °C–111 °C	Relatively low thermal gradients Barely feasible	[8, 9, 21]
	Panchagarh districtt				
	Salbanhat				
Rangpur saddle and the garo-rajmahal gap	Rangpur district	35 °C–47 °C	45.7 °C–115 °C	High surface temperatures Reasonable drilling depth	[9, 14, 21]
	Thakurgaon				
	Dinajpur district				
	Malda				
	Barapukuria				
	Western part of the Rangpur saddle				
Bogra slope	Singra	27 °C–31.6 °C	52 °C–147.8 °C	Potentially favorable	[14, 19, 21]
	Bogra				
	Kuchma				
Deepsedimentary basin	Madhupur	16.8–20.1 °C (Lower temperature gradient)	39.5 °C–104 °C	Loaded with cool sediment Geothermal gradients are very low Barely feasible	[14, 19, 21]
	Pabna				
	Mymensingh				
	Chandpur high				
	Barisal				
	Faridpur trough				
	Sylhet				
Folded Belt	Sitakund-5 Bakhrabad	23.0 °C	49 °C–118 °C	Geothermal gradients relatively low Feasible	[19, 21]

## 3 Demonstrated technologies to convert geothermal energy

Nature offers geothermal energy in different forms, namely, Geo-pressured brines, magma, hot, dry rocks, hydrothermal fluid and ambient ground heat [24]. However, the whole process in the utilization of geothermal energy can be divided into Exploration and Extraction as described in the following sections.

### 3.1 Exploration

**Figure 6:**
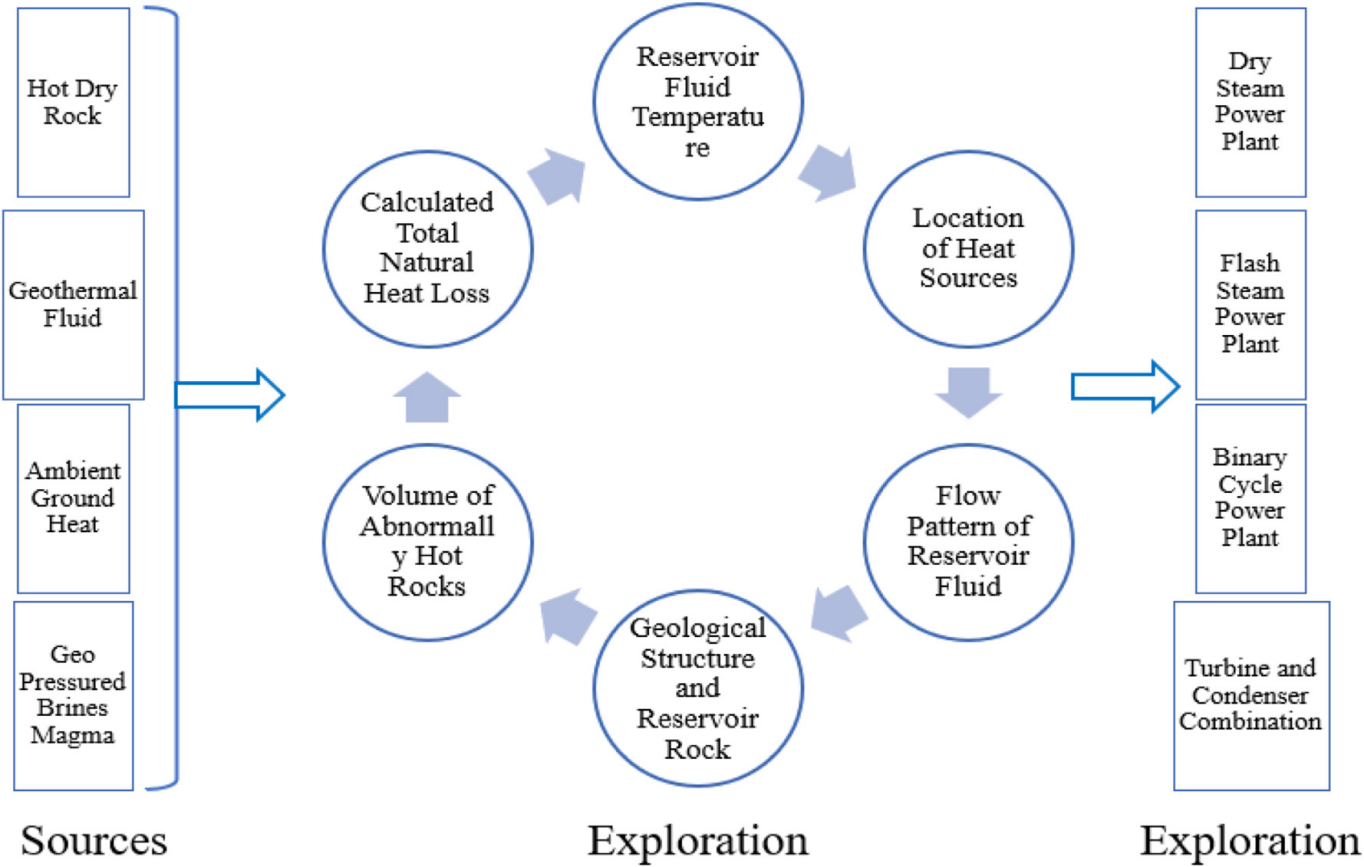
Geothermal energy lifecycle [21, 22, 23, 24, 25].

A concept of the total life cycle of geothermal energy is illustrated in Figure 6 [21,25].

In geothermal systems, physical parameters like temperature, pressure, porosity, chemical content of fluid, and permeability are the most important [26]. With current conventional geophysical methods, it is challenging to measure all these parameters although measuring temperatures, electrical resistivity, thermal conductivity, seismic velocity, density, and magnetization is more easily achieved [27]. The methods used for geothermal exploration include geochemical sampling and analysis, geological mapping, geophysical methods, and exploration drilling [26]. An integrated approach is used before proceeding to the most costly and critical process which is drilling [26]. An approach consisting of all of the above processes establishes a conceptual model of a subsurface geothermal system. After establishing a potential location, the following processes (Geology, Geochemistry, and Geophysics) are generally followed.

In the geology phase, a detailed geographical map is generated considering geothermal fluids and their surroundings, surface alteration minerals, and tectonic features. Also, ground-water levels along with a detailed mapping of groundwater, lakes, and springs should be conducted. Samples of thermal water, gases, and steam are generally analyzed for chemical elements (major and trace elements) and stable isotopes (oxygen, hydrogen, carbon, and sulfur) in the geochemistry phase.

In the geophysics phase, a range of different methods and applications are enlisted [26, 27]. The thermal method is the direct measurement of temperature, heat, and properties of a geothermal system. It includes measurement of soil temperature, detailed geothermal surface mapping, temperature measurement in 20–100 m gradients, airborne infrared, and local heat flow surveys. The electrical method is also known as the electrical resistivity method. Among the two-resistivity surveys, a shallow survey is performed with central-loop transmission electron microscopy (TEM) soundings or audio-magneto telluric (AMT) soundings where direct-current (DC) methods can also be used, whereas the deep survey is performed by magnetotelluric (MT) soundings. Gravity measurements are used to detect a difference in density among geological formations. Different gravitational forces result from different densities and are measured in mGal (milligal) or 10-3 cm/s^2^. This method can be used for discovering heat sources in volcanic rock. Magnetic measurements locate hidden intrusions, estimate their lengths, trace buried dykes and faults, and find areas of degraded magnetization caused by thermal activity. For measuring the distribution of sound velocity, anomalies existing in the earth, and attenuation of the sound wave, seismic methods are used. With this method two types of waves are generated, a P wave that travels in a similar direction of the wave and a slower S wave that travels perpendicular to the wave direction. A compilation of all these features establishes a conceptual model of the geothermal system enabling the most promising areas for production to be determined.

### 3.2 Extraction

Generally, energy is extracted by supplying generated hot steam to a turbine and thus producing electricity [26, 27]. Geothermal power plants mainly consist of three developed configurations, Binary Cycle power plant, Dry Steam, and Flash Steam. Amongst dry steam power is the simplest and oldest system where steam having 150 °C or more is used to turn the turbines. For this system, two wells are needed for rising the steam and injecting the water. Water is injected, which contacts the high-temperature rocks and produces steam that is pumped through the production well. The water is recycled in a closed-loop system. In flash steam power systems, hot and highly pressurized water is pulled into a lower pressure tank by using flash steam stations to run the turbine. This requires temperatures of ≥180 °C and produces electricity in a more efficient way than the dry steam process. In a binary cycle configuration, the vapor is produced by transferring heat into a low boiling point organic fluid. The advantage of this type of power plant is that it can work at reduced temperatures [28]. The typical efficiency lies within 10–13% [28]. It is considered as the most developed and efficient geothermal power plant, and here the efficiency is directly proportional to the temperature (up to about 180 °C). For water as fluid and reservoir temperature >220 °C flash systems or combined binary and flash systems are used. The binary cycle is favored for use when the temperature is within 100 °C–220 °C. At temperatures of 50 °C–150 °C, heating rather than power generation is an option. However, the binary cycle can also be used [21].

## 4 Comprehensive proposal

Global renewable energy resources are yet to achieve commercial acceptance, hence are currently a limited substitute to conventional energy resources. Nevertheless, they can provide a reasonable supplement to the long-term energy needs globally; ultimately improving the standard of living across the world. To date, hydro, solar, wind, biogas, and biomass have been used for the renewable energy sectors, although geothermal resources have had very little recognition, systemic exploration, and subsequent investment [6, 7, 8, 9, 14]. The importance of exploring safe, clean, and sustainable sources of energy is crucial, and the government is taking various steps to promote this initiative, namely facilitating investments across both private and public sectors in renewable energy projects. The current policy targets a 10% contribution from renewable resources to the total power generation by 2020 [29]. Among the objectives of these policies, the following are noteworthy:

1. Accelerating the uses of renewable energy for electricity production and heat generation.
2. Ensuring the optimum utilization of available renewable resources across rural, peri-urban, and urban areas.
3. Replacing conventional energy supplies through developed sustainable energies.

**Figure 7:**
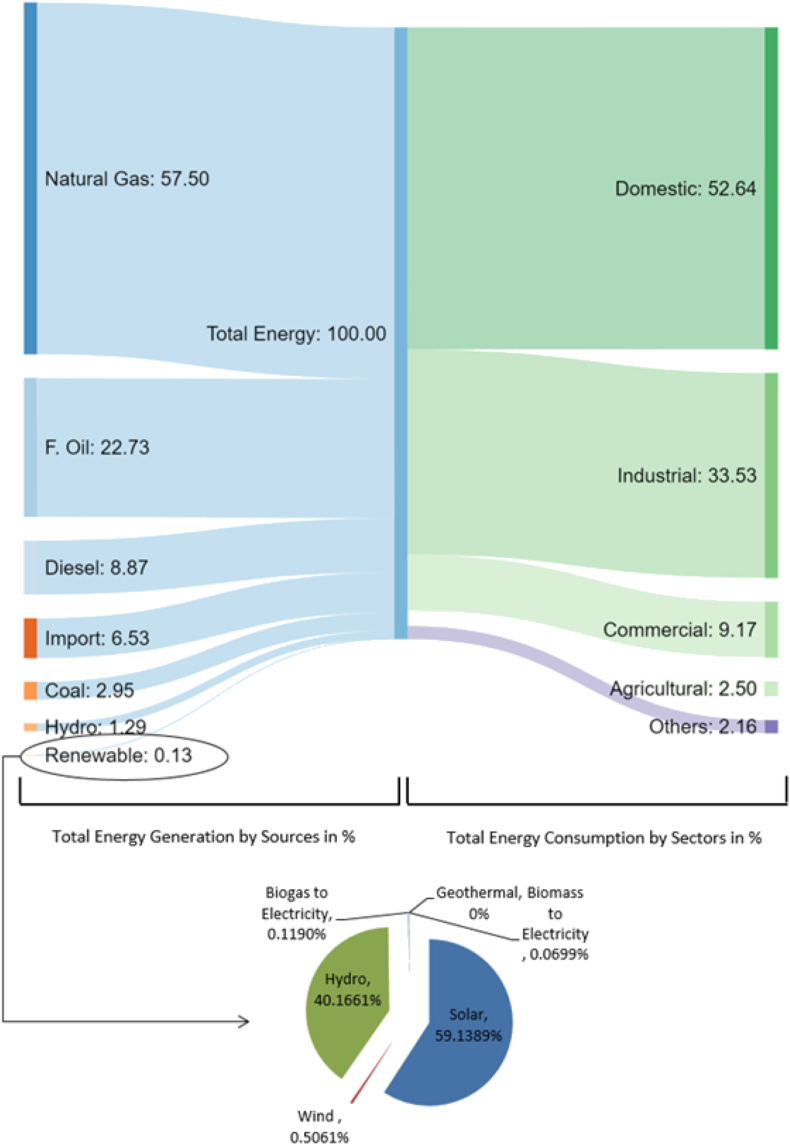
Primary energy consumption in Bangladesh by source and sector in 2019 [29, 30, 31].

**Figure 8:**
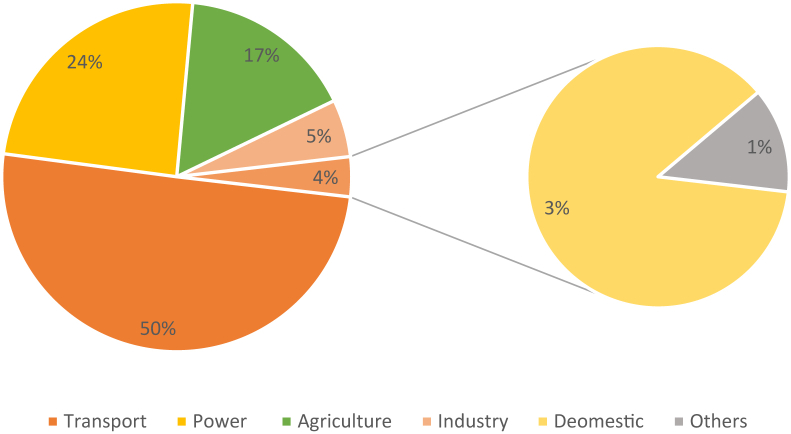
Sector-wise liquid fuel consumption in Bangladesh in 2018–19 [32].

These objectives directly promote and encourage the importance of evaluating geothermal resources and further broaden the scope of utilization. At present, it is estimated that ∼572.63 MW of energy (or 0.13% of total generation) is generated from renewable resources and is balanced between solar (59.138%), hydro (40.1661%), wind (0.5%), biomass (0.1%) and biogas (0.06%) (Figure 1). An increasing trend in solar projects is bolstering the contribution to total generation, but the lack of appropriate land and limitations in delivering consistent base-load energy constraining development in this sector. Additionally, Bangladesh has been ranked as the lowest hydro-power producing country in Asia, producing only about 230MW of electricity from hydroelectric power plants [30]. From Figure 7 the flow of energy production and utilization highlights a supply and demand scenario. Only a fraction of energy (572.63 MW) is produced utilizing renewable resources, which is around 0.13% of the total 17,764 MW production capacity [31]. Furthermore, it is evident from Figure 7 that Bangladesh largely depends on liquid fuel in its total energy generation, most of which is imported. Every year it imports about 1.36 million and 6.7 million metric tons of crude oil and refined petroleum products respectively. Although the major portion of its use is in transportation a considerable percentage is used across agriculture (24%) and industrial purposes (17%) as is illustrated in Figure 8 [32]. Transport is fundamental because it allows people to communicate, trade, and engage in other forms of exchange, which helps to develop civilizations. Furthermore, transportation is essential to economic development and globalization. Bangladesh, as a developing nation, seized this transportation opportunity quickly. As a result, transportation consumes a significant portion of the fuel. Bangladesh's primary energy sources are oil, gas, and coal. It is critical to secure primary energy sources to ensure power generation. Bangladesh's overall electricity demand in 2021 will be around 20,000 MW. To attain such a goal, relevant sectors must be assessed and stimulated by implementing short-, medium-and long-term plans. Fuel consumption is rising as a result of increased energy generation. The huge expenses associated with energy imports, particularly for agricultural and industrial purposes could be mitigated by adopting geothermal energy resources. Hence there is a dire need to incorporate the renewable energy matrix through an emerging source such as geothermal.

**Figure 9:**
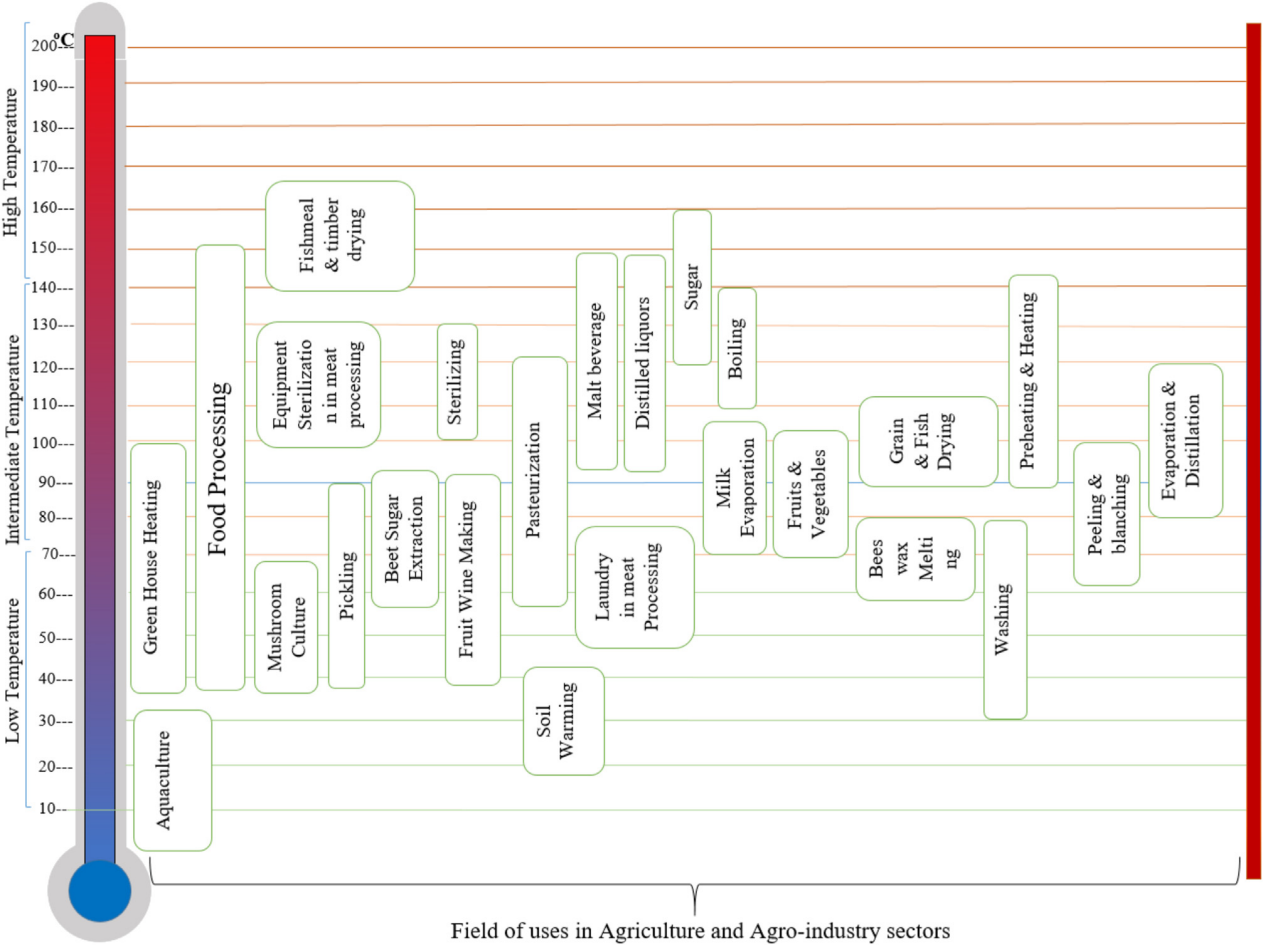
Geothermal advanced utilization according to the required temperature [34, 38, 39, 40, 41, 42, 43, 44, 45].

Figure 9 summarizes the potential uses of extracted energy from geothermal resources in agriculture and agro-industry sectors. In between low to an intermediate temperature (20 °C–150 °C), geothermal energy can be utilized in a direct application that includes space heating, heat pumps for heating and cooling, aquaculture, and various industrial processing systems. For direct electricity and steam generation temperature resources (70 °C–300 °C) are preferred. According to Bakos et al. [33], geothermal resources play a vital role in agro-industry and agricultural applications. These applications can be categorized into four sectors including greenhouse heating, agro-industrial process, aquaculture (fish and algae production) and soil heating. However, in agricultural and agro-industrial processing, geothermal resources only require low to intermediate temperature ranges, hence exploiting the waste heat and cascading water from a geothermal power plant are feasible [34]. For instance, drying technology is a critical process spanning many agricultural sectors to ensure the availability of food with high nutrition and in avoiding wastage. In this case, using geothermal resources having low to medium enthalpy with temperature less than 150 °C, and/or latent heat from geothermal power plants and hot well water would be a more cost-effective application [35, 36, 37].

**Figure 10:**
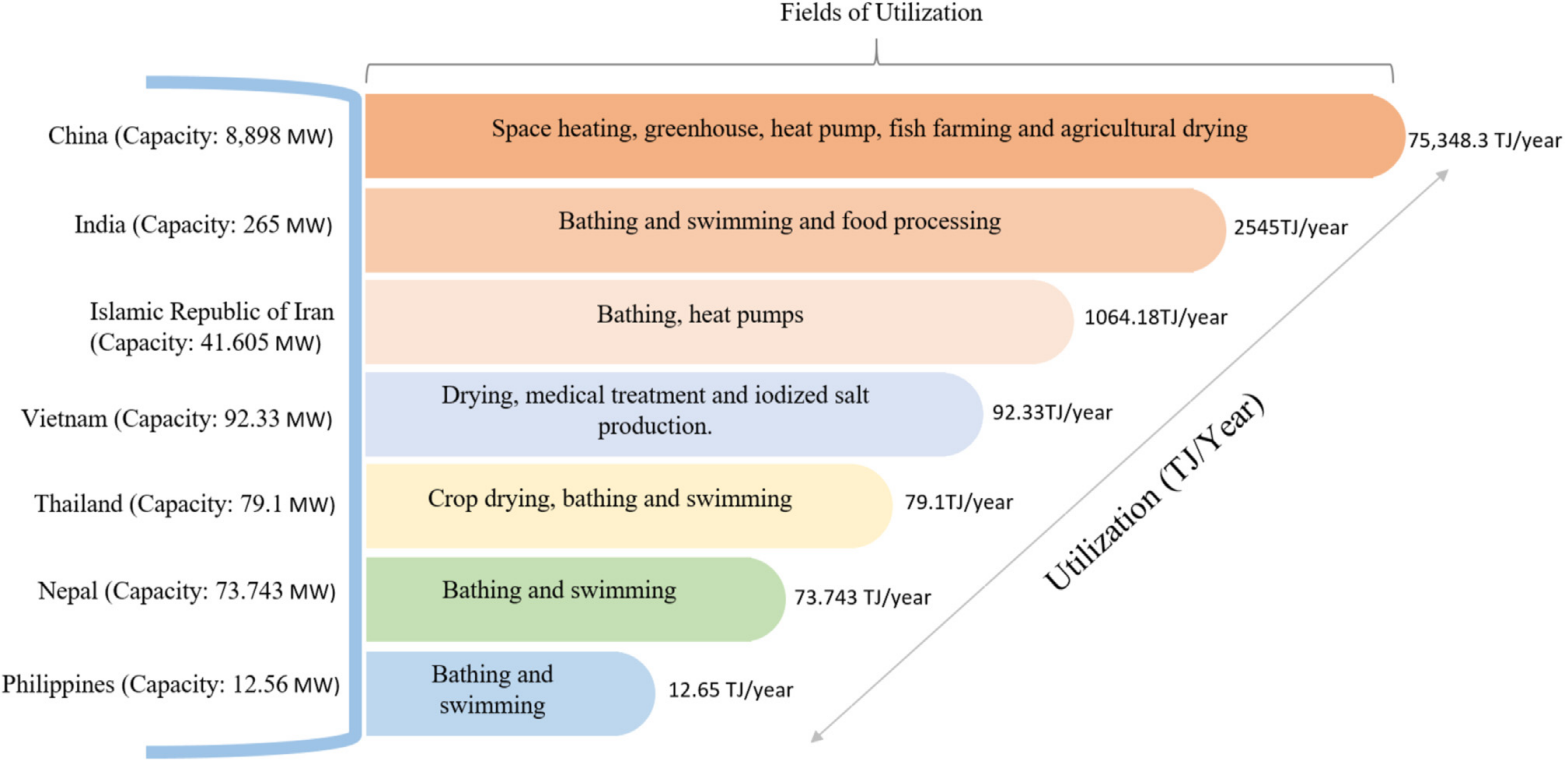
Comparison of the utilization of geothermal energy in Asian Countries [42, 46, 47, 48, 49, 50, 51].

As shown in Figure 10, it is evident that neighboring countries like India, China, and Nepal are already utilizing their geothermal resources in agriculture sectors like fish farming, drying along with industrial applications including space heating, heat pump, and in the domestic sector for bathing and swimming.

From the analysis of global geothermal utilization under similar temperature gradients, some plausible geothermal energy utilization processes that can be applied to Bangladesh are presented. The uses of geothermal energy can be categorized into direct use and indirect use. In indirect use, geothermal energy is used to produce electricity via dry steam, flash steam, or binary cycle power plants. For direct use, the most promising utilization is in agricultural drying, industrial application, aquaculture, and irrigation.

### 4.1 Agricultural drying

The drying of agricultural products is very important and would play a significant role in developing countries. It avoids wastage and ensures nutritious food year-round and during natural disasters and droughts. The recovered waste heat from the geothermal power plant, steam or hot water from the geothermal well can be utilized as the heating sources [37]. Around the world, different types of agricultural products are dried using this process. An agricultural drying plant in Nea Kessani Xanthi in Greece uses 59 °C geothermal water to commercially dry tomatoes [10]. Other examples of utilizing waste heat recovered from geothermal power include chilli and garlic drying at 50 °C and 70 °C respectively. In Thailand and the drying of pyrethrum [52], tobacco and maize in Kenya [53].

Rice is a staple food globally so the application of this technology in rice drying would be beneficial to any country's major agricultural sectors. The application in rice drying has been developed in Kotchany, Macedonia, and can serve as a model system for deployment. In this plant, the drying of rice is conducted directly from hot (78 °C) geothermal water and has a drying capacity of 10 tonnes/hour and a heat capacity of 1360 kW [54]. Fruit drying has been developed in Mexico with a capacity of 1 tonne of fruits per drying cycle and produces fruits with 20% moisture content within 24 h at 60 °C [55]. Indonesia possesses the highest potential for geothermal resources in the whole world [56, 57, 58]. Geothermal energy is considered a wise option to dry several of the crops produced in this region, such as coffee, beans, tea, rough rice, and fishery products [59]. In the Kamojang geothermal field of West Java, a special dryer operating at 160 °C has been established to dry beans and grains [60].

### 4.2 Industrial applications

**Figure 11:**
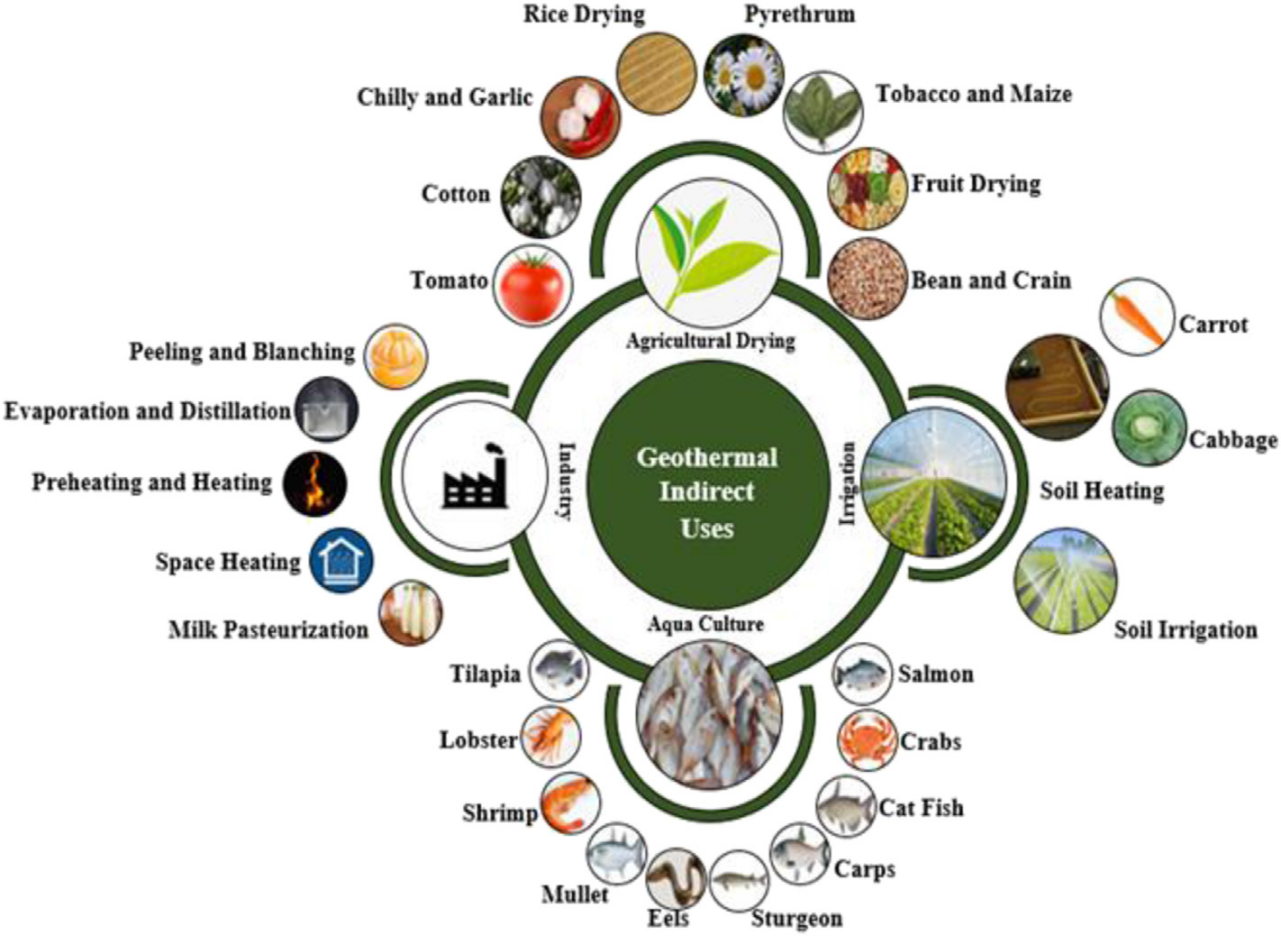
Direct means of geothermal energy utilization.

Industrial application of geothermal energy is broad reaching across many processes like milk pasteurization, preheating and heating process, evaporation, distillation, pealing, blanching foods, and general sterilization. These industrial processes are widely used in many countries and represent an optimistic way toward further utilization of geothermal energy [25]. Food processing industries have wide applications of preheating and heating and can utilize geothermal steam and geothermal hot water in the range of 90–150 °C [25]. In many industries like sugar mills, mint distillation, and liquor processing, evaporation and distillation are commonly used to concentrate the products and commonly operate at processing temperatures between 80 °C to 120 °C. Food processing industries have a very important pre-processing step (peeling and blanching) that requires temperatures from 77 °C to 104 °C [39]. Higher temperature (>120 °C) applications of geothermal hot water or steam can be used in the sterilization of food processing equipment, canning, and bottling industries [25, 39]. Figure 11 depicts the possible direct applications of geothermal energy.

### 4.3 Aquaculture

Aquaculture is a very important industry, especially for countries where rivers and fish are their major food commodity. Geothermal hot water is used to obtain suitable temperatures for aquaculture farming by mixing with cooler freshwater or using heat exchangers. This application of geothermal energy broadens aquaculture activities into cold climates and at places where alternative heating sources are not available or are costly [61]. It also prevents the stock from adverse weather changes, enhances the rate of production, is generally a cheaper heating alternative, and can ultimately lead to a greater economic return [62]. The main aquaculture species raised are illustrated in Figure 11. Geothermal heating in aquaculture is a relatively mature technology but is rapidly expanding in many countries like the USA, France, Iceland, Greece, and New Zealand. Also, fish and seaweed drying are another example of a large-scale industrial application in Iceland. In Iceland, there are almost 20 companies that are engaged in drying fish by using the hot water and steam generated in geothermal systems [25]. Another promising industry using geothermal energy is the drying of pet food which has a production rate of about 500 tonnes annually [63].

### 4.4 Irrigation

Several developed and developing countries in the world are agriculture-based. Utilizing geothermal energy for irrigation may have significant productivity and economic benefits. Both soil heating and soil irrigation are two potential applications. The growing season can be extended by soil heating, and it aids in the process of keeping the soil temperature constant. Carrots and cabbages have been cultivated using this technique by introducing 60 °C water through a grid of corrugated polypropylene pipe maintaining the soil at about 20–30 °C [64, 65]. Factors affecting the soil temperature in this system are air temperature, surface heat transfer coefficient of the soil, inlet, and outlet water temperatures, depth of the pipes installed, the distance between the pipes, and the effective thermal conductivity of the soil. Geothermal water can also be supplied through a surface irrigation piping system that is buried under the soil. However, when using this system, water salinity and chemical composition must be carefully monitored to avoid damage to the plants and soil.

## 5 Effective geothermal energy management model

The global world largely depends on agriculture as about 45% of the world's population relies on it for their livelihood. In the case of Bangladesh, the agricultural sector is a major employer (almost 63% of the total population) and contributes about 19.6% to the national GDP [66]. In general land under cultivation is ever increasing as is the demand for food [67, 68]. This illustrates a trend of using the same land for multiple crops. Across the sector, the production of potato, pumpkin, brinjal, cucumber, cabbage, beans, tomato, radish, cauliflower, and many more are continuing to increase. Throughout 2018, Bangladesh produces ∼9,744,412 tonnes of potato, with a world sixth, out of approximately ∼477,419 ha of potato [69].

Again, Bangladesh is one of the world's leading inland fisheries producers. During 2003–2004 it had produced 1,646,819 tonnes of products, 455,601 tonnes from the marine catch, and a total of 914,752 tonnes from aquaculture. At the end of the year, total production reached above 2.1 million tonnes [70, 71]. FAO declared Bangladesh as the sixth-largest aquaculture-producing country by the year 2005. In the year 2003, it had produced 856956 tonnes [70]. During the year 2003–2004, about 43.5% of the total fish was accounted for aquaculture whereas the inland open water fisheries contributed about 34.8% [72].

**Figure 12:**
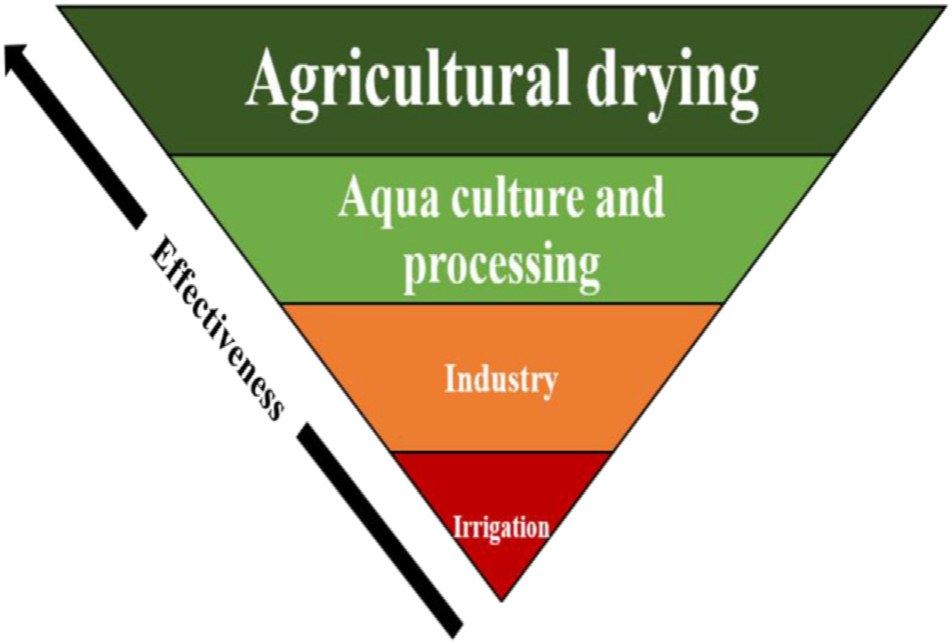
Effective geothermal management model.

The effectiveness of the discussed the direct application of various geothermal energy. Bangladesh is summarized in Table 2. Here, an effective geothermal energy management model is also developed considering the suitable conditions of Bangladesh. The direct uses of geothermal energy which are most suitable for Bangladesh are ranked in descending order of effectiveness in Figure 12 and suggest agricultural drying and aquaculture are the most likely ‘best bet’ applications in the short term. The effectiveness is ranked according to the operational time, implementation cost, and economic values of each sector. Bangladesh is predominantly considered as an agricultural country in which agriculture plays an accelerating role in economic development. Peoples living in this country, directly and indirectly, depend on it all year round. Also, being a tropical country and having fertile soil, it offers naturally favorable conditions for harvesting crops, vegetables, fruits, and spices. The economic value of this sector is comparatively higher than all other sectors and the implementation cost for geothermal energy extraction will not be so high as nature offers favorable weather conditions already. Again, in Bangladesh, the fisheries and the aquaculture industry lead an important role in mitigating malnutrition and protein deficiency. Almost 60% of the total animal protein consumption comes from fish industries [73]. This sector has also a great contribution to employment generation and foreign exchange earnings. However, during winter the water temperature in Bangladesh tends to drop below 15 °C which is not supportive for favorable production whereas for the highest production the recommended optimal temperature ranges between 22 to 30 °C [73]. For this, geothermal energy can easily be extracted for heating as the geothermal potentials of Bangladesh support favorable conditions in this regard which leads to a reasonable cost for implementation. Using geothermal energy for fish drying and aquaculture will also develop the industry and promote a renewable energy-based economy. Though the economic value of aquaculture sectors is minor than the industrial perspective, it costs comparatively lower than industry in terms of implementation. This is because industrial uses need comparatively higher heating facilities than aquaculture heating which results in drilling more depth for acquiring suitable temperature and leads to a higher cost. In Bangladesh, several irrigation techniques are also used such as – power pumps, tube wells, canals, etc. Geothermal resources can be used for soil heating thus maintaining constant ground temperature to increase yields. The soil temperature is maintained at about 20–30 °C. Geothermal water can also be used in irrigation keeping in mind the chemical composition and salinity of the water [74]. However, the effectiveness of using geothermal energy in other sectors is relatively the lowest in Bangladesh in terms of cost.

**Table 2:** Effectiveness of indirect uses of geothermal energy, Extracted from [75].

Application	Economic value	Operation time	Implementation Cost and Logistics
Agricultural drying	Potentially dryable 1.288 Mega Tons (Mt) of vegetables, 3.830 Mt of fruits and 2.594 Mt of spices	12 months	Low - Medium
Aquaculture and processing	4.14 Mt	6 months (winter season)	Low- Medium
Industry	81,645 tons of tea, 74,699 thousand soft drinks and cigarette, 891.424 Mt knitwear	12 months	High
Irrigation	18,820 thousand acres of land cultivable land	8–12 months	Medium - High

## 6 Geothermal plants

**Figure 13:**
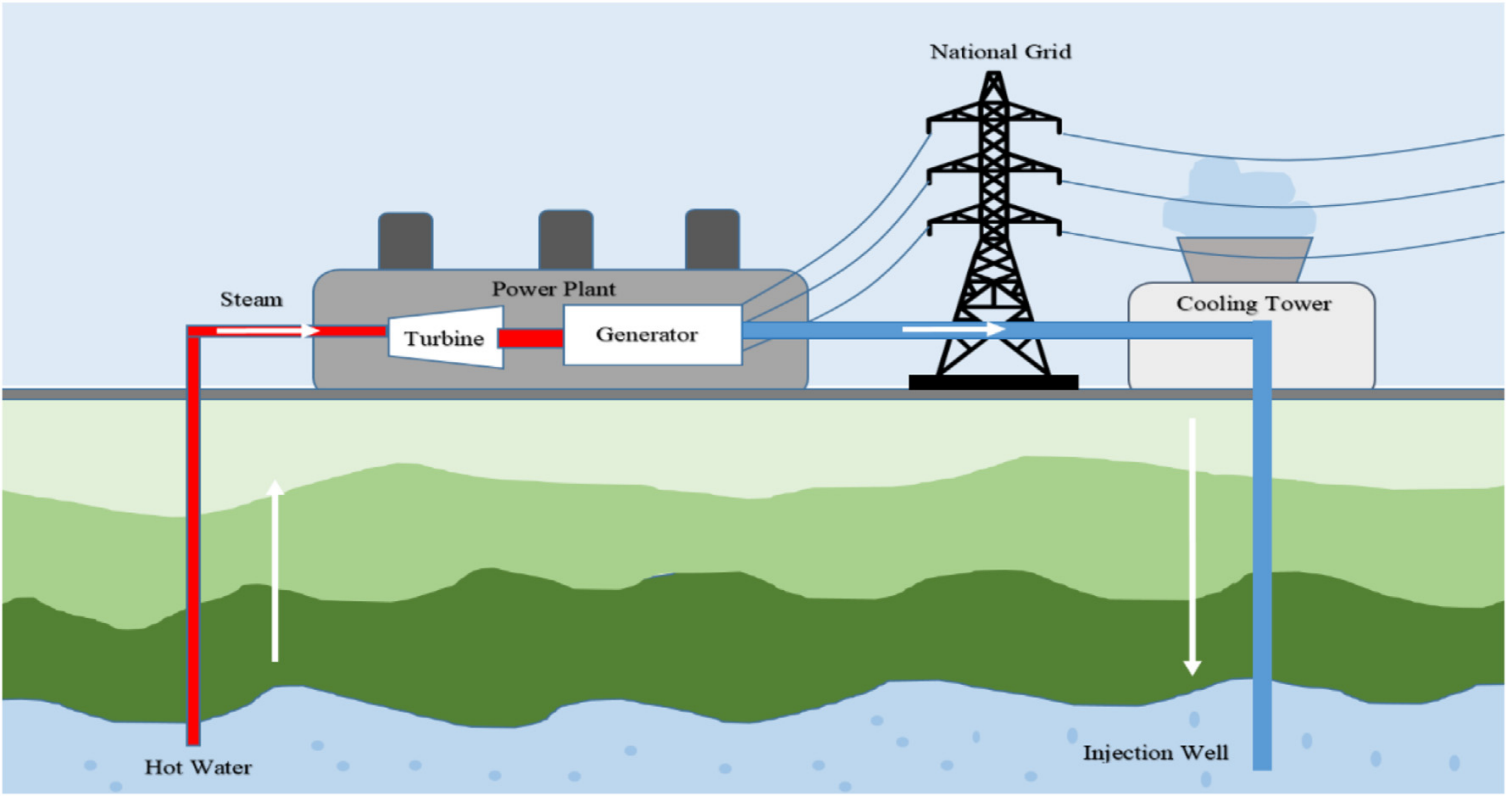
A flow diagram of geothermal energy extraction by a power plant [Ref].

It is well established that geothermal energy can be directly extracted as heat in order to produce electricity incorporating the principle of a steam power plant. Figure 13 demonstrates one such geothermal-based electricity production pipeline. In its simplest form, heat energy is captured using water which then, in turn, produces steam to drive a turbine-based electricity generation system. A range of different processes for extracting geothermal energy have been configured and are typically tailored to the geothermal temperature gradients of the region.

**Figure 14:**
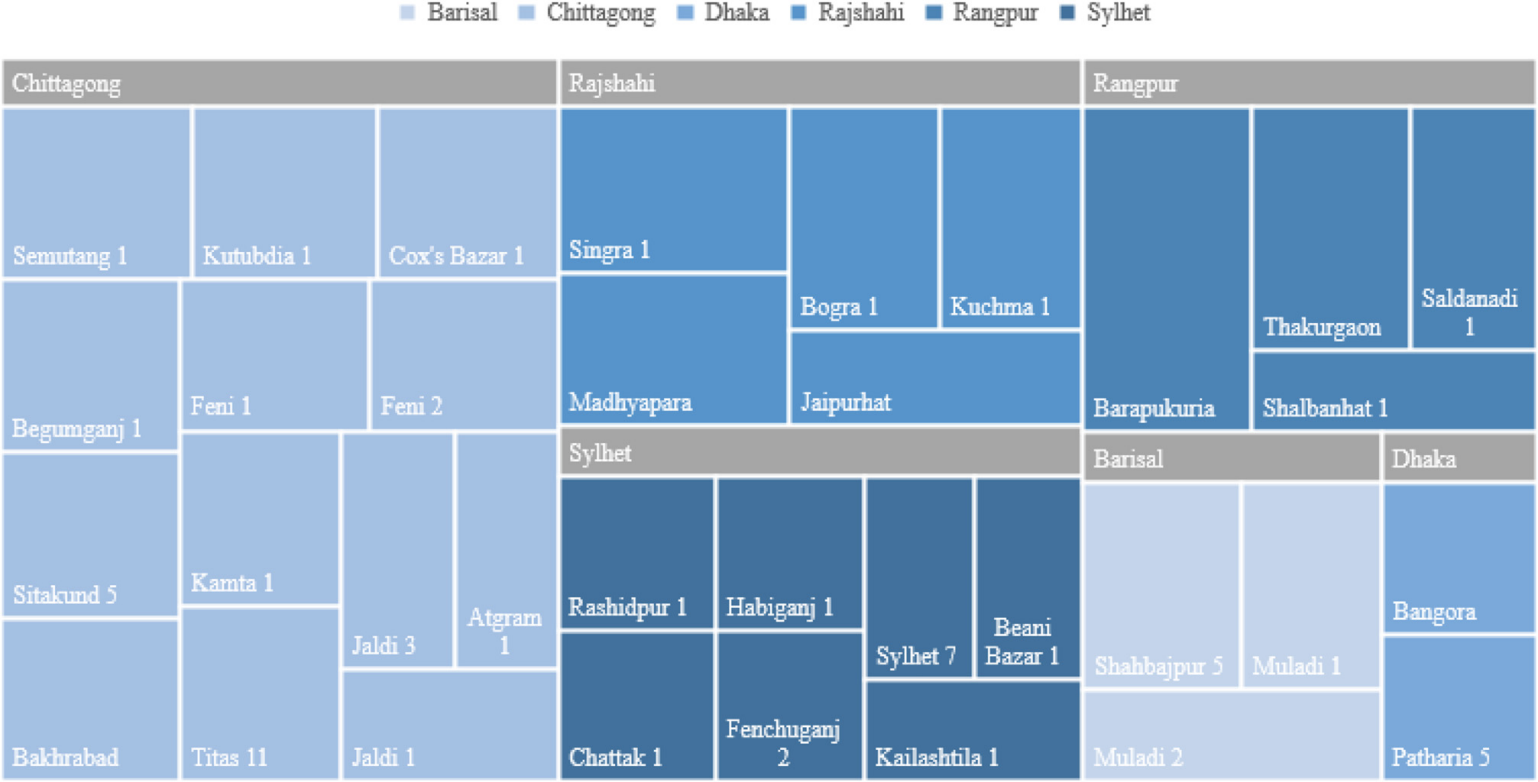
Tree map describing geothermal gradients in various divisions of Bangladesh.

As previously discussed, Bangladesh has several geothermal energy-rich locations with the potential to develop geothermal energy. Further analysis of the 62 exploratory wells dug in various regions of Bangladesh has been used to classify the range of geothermal temperature gradients. Based on this data it could be said that there are 6 divisions of Bangladesh in which geothermal-based energy conversion plants can be effectively and economically established. Figure 14 uses a tree map of geothermal-rich areas in Bangladesh classified into 6 divisions. The tree map denotes the geothermal gradient potential by area, where a larger area represents higher geothermal potential. From Figure 14 it is evident that the northernmost division Rangpur and Sylhet, the southernmost division Chittagong have the most effective geothermal potential, where Thakurgaon and Barapukuria of Rangpur division have the highest geothermal gradient. From these regions, fluid with high enthalpy can be supplied for the reservoir of a geothermal power plant. On the other hand, Barisal and Dhaka divisions along with low-temperature gradients compared to Rangpur and Chittagong regions. From Table 1 it is also found that Faridpur trough, Pabna, Madhupur, Chandpur also have relatively low-temperature gradients. These areas are not considered suitable for developing geothermal power plants since the cost of drilling is very high.

**Figure 15:**
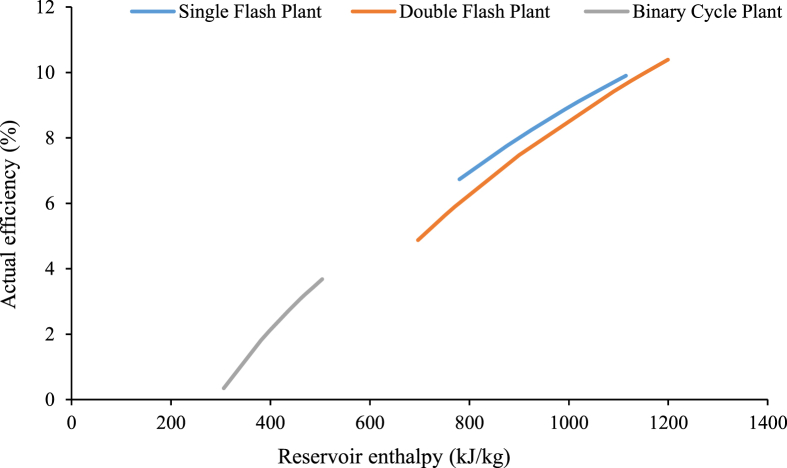
Geothermal Power plant efficiency comparison concerning the enthalpy of the reservoir fluid.

From the previous discussion, it is seen that there are mainly three types of geothermal power plant which are used worldwide for energy generation (Dry cycle, Flash steam, and Binary cycle). Flash steam can be further divided into two process configurations, Single flash, and Double flash. Technically the Single flash and Dry steam are similar processes and employed when the field is dry [76]. Among all the different processes, the Binary cycle power plant is the most developed power plant which can operate at a lower temperature with 10–13% efficiency [76]. However, as the temperature increases, process efficiencies are improved [21] as indicated in Figure 15. The efficiencies presented in Figure 15 were measured based on the enthalpy (*h*) of the reservoir's fluid of different power plants and calculated using (1), (2), (3) [76].

For Single Flash Plant,

(1) $$\eta_{\text{act}}=8.7007\;\ln\left(h\right)-52.335$$

For Double Flash Plant,

(2) $$\eta_{\text{act}}=10.166\;\ln\left(h\right)-61.68$$

For Binary Cycle Plant,

(3) $$\eta_{\text{act}}=6.6869\;\ln\left(h\right)-37.929$$

A study by Moon et al. (2014) [76] provides a result of Binary plant which can generate electricity from water temperatures of 73 °C (306 kJ/kg). However, the combined binary-flash system can be adopted at a higher temperature (above 180 °C) and using water or steam as the reservoir fluid. On the contrary, when water reservoir fluids have temperatures between 100 °C and 220 °C, a binary cycle power plant is a better option [77]. A summary of different geothermal power plants around the world showing process configuration and enthalpy of reservoir fluids is given in Table 3.

**Table 3:** Different types of installed geothermal plants around the world.

Type	Country	Plant Name	Installed Capacity (MWe)	Reservoir Fluid Enthalpy (kJ/kg)	References
Single Flash Type	Russia	Pauzhetka	11	780	[78, 79]
	Turkey	Kizildere	20.4	875	[76, 79, 80, 81]
	Japan	Oita (Takigami)	25	925	[80, 82]
	Japan	Akita (onuma)	9.5	966	[80, 82, 83]
	Japan	Iwate (kakkonda)	80	992	[82, 84, 85]
	Japan	Miyagi (Onikobe)	12.5	1020	[82, 83, 86, 87]
	USA	Utah-Roosevelt Hot Springs (Blundell1)	26	1062	[76, 88]
	Costa Rica	Miravalles	144	1107	[83, 89, 90]
	France	Bouillante 2	11	1110	[91, 92]
	El Salvador	Ahuahapan (U1,2)	60	1115	[93, 94]
Double Flash Type	USA	California-East Mesa (GEM2,3)	37	697	[95, 96, 97]
	Mexico	Cerro Prieto (CP-1, Unit 5)	35	742	[98, 99, 100]
	USA	Nevada	26	750	[101, 102]
	USA	California-Heber	52	771	[95, 96]
	USA	Nevada	16.7	900	[103, 104, 105]
	El Salvador	Ahuahapan (U3)	35	1091	[93, 94]
	France	Bouillante 1	4	1092	[42, 114]
	Japan	Oita (hatchobaru)	110	1125	[80, 95, 105, 106]
	New Zealand	Reporoa (Ohaaki)	46.7	1150	[76, 80]
	Japan	Mori	50	1199	[82, 107, 108]
Binary Cycle Power Plant	USA	Alaska (Chena Hot Springs)	0.5	306	[76, 109]
	USA	Wyoming-Casper (Rmotc-Ghcg)	0.25	381	[110, 111]
	Germany	Neustadt-Glew	0.23	398	[112, 113]
	USA	Nevada	2.2	436	[76, 103]
	Australia	Altheim	1	444	[114, 115]
	Australia	Blumau	0.2	461	[114, 116, 117]
	USA	California-Honey Lake	0.7	461	[103, 118]
	China	Nagqu	1	470	[80, 119]
	Thailand	Fang	0.3	487	[80, 114]
	Germany	Unter-Haching	3.36	504	[112]

It is revealed from Table 3 that the binary cycle power plant can operate at considerable efficiency with reservoir fluids of lower enthalpy compared to Flash configurations. It is also worth noting that the installed capacity (Megawatts, MWe) of current Binary cycle plants is significantly lower than Flash-based systems (Refer to Table 3). Based on the temperatures of Bangladesh's highest potential areas, it suggests that the Binary cycle systems would be the most favorable operational system for deployment. Furthermore, since the single and double flash power plant work on relatively higher temperatures, implementing these systems across Bangladesh's geothermal regions would be a more costly approach due to the necessity of deeper wells. The cost-benefit of delivering systems with a greater installed capacity based on accessing reservoirs of higher potential enthalpy warrants further investigation.

## 7 Effective cost analysis for a establishing a geothermal plant

The cost for establishing a geothermal plant can be predicted by analyzing the capital cost, operation and maintenance cost, and equipment cost during installation, commissioning and power generation. Each cost depends on the project requirements such as turbine, air-cooled condenser, PEM electrolyser, water heater, and many more. Furthermore, the cost range depends on the type, well productivity, and geothermal power plant's geothermal economic parameters.

Initially, to evaluate economic viability, the estimated capital costs for geothermal plants are required. Because the capital cost is fixed, the one-off costs are incurred in purchasing the land, buildings, and equipment used to produce goods or render services. A geothermal power plant's capital cost can be ranged between 1,092$ and 347,100$ (Table 4), where turbine and PEM (Polymer Electrolyte Membrane) electrolyser cost is higher than other equipment. Hydrogen production quantities are negligible at low current density, resulting in a very significant capital cost per unit of hydrogen produced. For this reason, the capital cost of the PEM electrolyser is intensive. It would be the next concern for further research in the future. According to Table 4, the maintenance and operational cost are ranged between 58.50 and 713,700$, in which the highest price is carried out by an air-cooled condenser. The air-cooled condenser cost depends on ITD (Initial Temperature Difference). Cost of an air-cooled condenser increase with decreasing ITD [120]. Nevertheless, the purchase cost and specific exergetic cost are maximum for the PEM electrolyzer. Consequently, from Table 4, it can be concluded that PEM can be the primary concern regarding price for further research.

**Table 4:** Summary of cost analysis for different categories with related equations.

Categories	Area of investment	Cost ($)	Cost range ($)	Required equations
Capital cost [121, 124, 125]	Turbine	56,589	1,092–347,100	• $A=CRF\sum_{m=1}^{n}P_{m}=\frac{i_{eff}{\left(1+i_{eff}\right)}^{n}}{{\left(1+i_{eff}\right)}^{n}-1}\sum_{m=1}^{n}P_{m}$ Here, $P_{m}=C_{m}\frac{1}{{\left(1+i_{eff}\right)}^{m}}$ • $Z_{k}^{T}=\left[\frac{CC_{L}+OMC_{L}}{\tau }\right]\frac{PEC_{K}}{\sum_{K}PEC_{K}}$
	Air cooled condenser	13,650		
	Pump	8,970		
	PEM electrolyzer	347,100		
	Heat exchanger	18,018		
	Water preheater	5,460		
	Other equipment	1,092		
Operation and maintenance cost (OM) [125]	Turbine	2964	58.50–713,700	
	Air cooled condenser	713,700		
	Pump	468		
	PEM electrolyzer	18,135		
	Heat exchanger	956		
	Water preheater	293		
	Other equipment	58.50		
Purchased equipment cost [121]	Turbine	1066.700	20,500–6,531,300	• Based on Aspen Plus economic analysis library [121]
	Air cooled condenser	257,300		
	Pump	168,700		
	PEM electrolyzer	6,531,300		
	Heat exchanger	339,600		
	Water preheater	102,700		
	Other equipment	20,500		
Energetic and exergetic cost [125]	Pump	13.58	2.246–19.68	• Specific cost of the exergy of product, $c_{P}=\frac{\dot{C_{P}}}{{\dot{Ex}}_{F}}$
	Heat exchanger	2.246		
	Turbine	6.496		
	Air cooled condenser	4.234		
	PEM electrolysis	19.68		
	Electrolysis water preheater	5.475		
Economic parameters [126]	Surface cost	45.75 × 10^6^	–	• $C_{surface}=C_{1}.\exp\left(-0.0045\;\left(\dot{W-C_{2}}\right)\right)$ • $C_{OM}=C_{1}.\exp\left(-0.0025\;\left(\dot{W-C_{2}}\right)\right)$
	OM cost	3755.33		
	Subsurface cost	45.76 × 10^6^		
	Initial cost	91.52		
Macro-economic [126]	Fuel cost escalation rate (%)	4.5	–	–
	Debt interest ratio (%)	8		
	Electricity export rate	91.3		

However, the general and macro-economic parameters must use to measure the geothermal plant's cost, which can be obtained from the equation mentioned in Table 4. In economic parameters, the surface and subsurface cost is so much higher than other parameters. Chamorro et al. [121] mentioned that the total initial cost is partially proportional to surface cost and subsurface costs. In terms of surface costs, the cost of unit capital is reasonably assumed to decrease exponentially due to economies of scale with an increasing plant capacity. The unit capital also depends on the technology used, increasing the complexity of the used technique. The author [121] used the following equation to measure a 5 MW geothermal plant's surface cost, and claimed that geothermal energy plant investment costs are susceptible to specific local characteristics:

(4) $$\left(C_{surface}=C_{1}.\exp\left(-0.0045\;\left(\dot{W-C_{2}}\right)\right)\right)$$

where, W = Installed power capacity, MW, $C_{surface}$ = Surface equipment unit cost, $/kW.

From the estimation of the author [121], it can be seen that the cost estimation calculated based on 5 MW power plant. According to the Bangladesh Power Development Board [122], the average cost range of all power plants (2–970 MW) in Bangladesh is 22 × 10^6^ dollar to 320×10^6^ dollar. But from the above description, it can be seen that the cost range of a 5 MW plant is less than other plants in Bangladesh. In 2010, the Rahimafrooz group signed up a 5 MW power plant in Bangladesh, with an estimated installation cost of 25 million [123]. The installation cost is less than all other plants in Bangladesh. From this, it can be said that the geothermal plant can be a promising solution for the future. And it is essential not only for cleaner production but also for low-cost power production. Besides from the above description, it is suggested that further research can be focused on the techno-economic analysis of the geothermal plant. Furthermore, exergetic cost optimization can be considered for better outcomes. Exergetic optimization of the system is a method of reducing exergy destruction. This analysis can provide optimal parameters to minimize exergy losses and exercise costs.

## 8 Bounds of applicability

It is important that the utilisation of new/renewable resources does not create further environmental or societal problems. For Bangladesh, geothermal resources can be an auspicious source of energy and a new opportunity to further increase the contribution of renewables to the total energy conversion. However, there are some impediments in the path of complete utilization of geothermal energy which is stated below:

### 8.1 Environmental concerns

#### 8.1.1 Surface disturbance and physical effect of fluid withdrawal

Surface disturbances occur during drilling. Generally, an area covered by a drill site range from 200-2500m^2^ and the well are kept to a minimum by directional from a single site. Additionally, evidence of bad landslides have been found which were connected directly to the installations of geothermal plants [127].

Again, fluid withdrawal has far-reaching effects such as affecting surface manifestations, the disappearance of hot springs or geysers, or transformation into fumaroles [128]. A serious disaster like lowering groundwater labels, subsidence of land, and induced seismicity can happen. Also, for lowering of the groundwater results in mixing the fluids of aquifers which causes an inflow of corrosive water. Some other effects like accelerating the growth or formation of the steam pillow, consequent boiling, and degassing of the field will lead to major explosions killing several people in the past [129].

#### 8.1.2 Noise and thermal effects

Construction (e.g., drilling) and pumping/process noise can be significant. However, established techniques exist to control this to acceptable levels [130, 131, 132, 133, 134]. Moreover, because of the lowering efficiency of power production, thermal effects cause energy wastage. An excessive amount of heat coming out as steam results in cloud formation hence causing a drastic change in the weather and wastewater directed to the stream, rivers, or lakes may cause a biological and ecological impact on this area.

#### 8.1.3 Chemical pollution

Chemical pollution takes place due to the discharge of water or steam into the environment which may contain chemicals. Heavy metals are of particular concern, as well as hydrogen sulphide (H_2_S). Bangladesh has a history of severe arsenic poisoning due to contamination of drinking water which has not yet been fully resolved [135]. Testing wells and altering well depths have partially solved this issue in some regions. Care must be taken to ensure that the utilization of geothermal energy does not contaminate drinking water supplies with high arsenic water extracted from different depths or regions to the well. Disposal of water containing brines may be very risky as As and Hg may accumulate in organisms and sediments also a high concentration of boron is also harmful to plants thus causing direct damage to the environment. The treatment being an option becomes considered rarely for economic viability. Air pollution is also a problem caused due to the geothermal gases in steam. Carbon dioxide, hydrogen sulphide, methane, mercury, radon, ammonia, and boron are the common geothermal gases. Hydrogen sulphide may be the greatest concern as it has an unpleasant smell and is toxic at moderate concentrations. Geothermal gases also have diverse effects on the biological aspects of an area.

#### 8.1.4 Protection of natural features

The protection of natural features is a must for any region. Phenomena such as geysers, silica sinter terraces and mud pools, hot springs, or pools, to deteriorate or disappear, along with special thermophilic vegetation such as algal mats, bacteria and thermophilic plants cause disturbances in the natural state [128, 136, 137].

#### 8.1.5 Policy and regulatory concerns

Few policies of the GoB declared the promotion of the use of renewable energy resources, however, to ensure more involvement of the renewable energy utilization, budgetary allotment should be increased as it tends to be low in developing countries [138]. Lack of financial resources is also an impediment, and the legislative framework is not enough to attract public, private, and foreign investment in geothermal projects. The Government can finance the early phases such as exploitation and appraisal through the right policy environment thus encouraging more geothermal initiatives to be in action. Developing countries lack the right institutional framework coordination among the corresponding stakeholders. These prevent the development of the implementation of these projects.

#### 8.1.6 Technical concerns and financial concerns

Expertise in the technical field, an expert mass of policy analysts, engineers, economic managers, and other professionals are necessary for developing geothermal systems. Shortage of qualified personnel in the first and basic need for a developing country. Transport systems and communication networks, as well as infrastructure to support geothermal systems, are insufficient. Moreover, high upfront cost often blocks the chances of new investors getting involved in geothermal projects. The shortage of funding discourages the investors from taking the first step, such as the assessment of the feasibility of geothermal energy. Financing is very important in the case of geothermal programs and challenges such as developing models to ensure sustainability and affordability often come in front. Financial institutions imposing infeasible conditions often become a disincentive to potential investors.

## 9 Conclusion

Geothermal energy has proven its capacity to be a reliable, clean, and proven source of providing uninterrupted sustainable renewable energy to societies across different economic and political backgrounds. More than 78 countries are currently using geothermal directly or indirectly, either for electricity production, industrial, agricultural, or domestic applications. Although Bangladesh currently does not have any active geothermal plants, it is considered a favorable location in Southeast Asia, a fact that has been established throughout this study. The main conclusions of this study can be summarized as follows:

a) In the global world there have been analysed from every possible aspect and have the potential to adopt sustainable and economically feasible geothermal energy technologies.
b) The major application of geothermal energy in Asia opts towards indirect use such as domestic water heating, heat pump, industrial drying, food drying, aquaculture, agriculture, and space heating. Bangladesh in its current development trend has the potential to adopt any such technology in its urban development framework.
c) Like developed countries in the world, it is also possible to establish any geothermal plant in developing countries as the reservoir fluid enthalpies of the potential areas are in the good range for implementing binary systems for power generation.
d) To reduce the energy crisis geothermal plants can be a promising solution for the world in the future and essential not only for cleaner production but also for low-cost power production. However, further research can be focused on the techno-economic analysis of the geothermal plant for better outcomes.
e) Despite the promise of geothermal energy as an effective alternative to fossil fuel, political dimension, and cheap non-renewable energy, a framework must be carefully analyzed and properly modified to improve geothermal energy projects globally.
f) In terms of utilizing geothermal energy, drying in the agricultural sector possesses the highest potential, and following it comes aquaculture and process, industrial use, and irrigation.

Currently, there is no exhaustive geothermal energy plan or legislation in Bangladesh. However, the “Energy Efficiency and Conservation Master Plan up to 2030” has provided strong incentive research and development of geothermal energy projects. In the absence of a strict renewable energy policy enforcing the law, the government of Bangladesh does not yet have any plan for the immediate adoption of geothermal renewable energy at a national scale. Amidst the policy of the heavily subsidized fossil fuel industry, the governmental and private investment in the renewable energy sector has fallen far behind its demand. In 2019 only 2.95% of national grid output was produced from renewable energy. There persists a severe shortage of up-to-date information and survey results on the geothermal potential in Bangladesh. Most of the research was performed using secondary data collected over a decade ago. This impacts negatively on potential foreign investment. However, it has come to a dire need to concentrate on strong research activities for updating the data and finding ways of harnessing this energy. Technical expertise and qualified personnel play a vital role in developing this system. A critical mass of engineers and other professional individuals like a geologist, geographers are required for this. However, continuing lack of professional individuals remains in our country because of proper technical training and the absence of studies regarding geothermal in almost all the technical institutions. Again, the majority are dependent on grid-connected electricity. Although there are many engineers, laboratories, technical staff, researchers but the development of geothermal is disreputable because of lacking government patronage, state funding, and proper knowledge. Thus, it is suggested to take these into consideration and task each technical institute to generate their electricity through favorable renewable energy. Moreover, essential training and courses should be provided to promote geothermal energy uses. These courses can contain the advancement of medium-low temperature geothermal power generation, solar-geothermal hybrid (SGH) power generation, geothermal integrated with combined cooling, heating, and power (CCHP) generation. Authorities should encourage to make prototypes and help in developing pilot projects. In addition, studies related to cost-efficient and scale improvement of geothermal power generation can be added.

## Declarations

### Author contribution statement

All authors listed have significantly contributed to the development and the writing of this article.

### Funding statement

None.

### Data availability statement

Data will be made available on request.

### Declaration of interest's statement

The authors declare no conflict of interest.

### Additional information

No additional information is available for this paper.
